# SLC7A11/GPX4 Inactivation-Mediated Ferroptosis Contributes to the Pathogenesis of Triptolide-Induced Cardiotoxicity

**DOI:** 10.1155/2022/3192607

**Published:** 2022-06-16

**Authors:** Xian Liu, Cheng Chen, Dong Han, Wei Zhou, Yaowen Cui, Xianglin Tang, Chengrong Xiao, Yuguang Wang, Yue Gao

**Affiliations:** ^1^Department of Pharmaceutical Sciences, Beijing Institute of Radiation Medicine, 100850 Beijing, China; ^2^The Second Medical Center & National Clinical Research Center for Geriatric Diseases, Chinese PLA General Hospital, 100853 Beijing, China; ^3^School of Pharmacy, Guangdong Pharmaceutical University, 5110006 Guangzhou, China

## Abstract

Triptolide exhibits promising efficacy in various cancers and immune diseases while its clinical application has been strongly restricted by its severe side effects, especially cardiotoxicity. However, the underlying mechanism of triptolide-induced cardiotoxicity (TIC) remains unclear. The RNA-seq analysis of triptolide-injured AC16 human cardiomyocyte cell line hinted that ferroptosis is involved in TIC. Further experimental validations proved that triptolide triggered ferroptosis, as evidenced by significant accumulation of lipid peroxidation (4-HNE and MDA levels) and ferrous iron, as well as depletion of intracellular GSH. Furthermore, triptolide-induced iron overload involved the upregulation of TF/TRFC/DMT1 signal axis and the degradation of ferritin, which contribute to ROS generation *via* Fenton reaction. In addition, inhibition of the antioxidant Nrf2/HO-1 pathway was observed in TIC, which may also lead to the overproduction of lethal lipid peroxides. Mechanistically, using streptavidin–biotin affinity pull-down assay and computational molecular docking, we unveiled that triptolide directly binds to SLC7A11 to inactivate SLC7A11/GPX4 signal axis. More importantly, employment of a ferroptosis inhibitor Ferrostatin-1 alleviated TIC by partially reversing the inhibitory effects of triptolide on SLC7A11/GPX4 signal. Altogether, our study demonstrated that SLC7A11/GPX4 inactivation-mediated ferroptosis contributed to the pathogenesis of TIC. Combating ferroptosis may be a promising therapeutic avenue to prevent TIC.

## 1. Introduction

Traditional Chinese herb *Tripterygium wilfordii* Hook F (TWHF, also known as Thunder God Vine) has been used for centuries in Asian to treat various diseases such as rheumatoid arthritis, lupus, nephrotic syndrome, and Behçet's disease [1]. Triptolide, as the major active component of TWHF, has exhibited potent antirheumatic, anti-inflammatory, immunomodulatory, antitumor, and antimicrobial effects and aroused interests from researchers for decades due to its great efficacy and remarkable clinical performance [2–4]. However, the clinical application of triptolide was limited by its extensive side effects on organs such as the liver, kidney, heart, testes and gastrointestinal tract [5, 6]. The clinical case reports indicated that exposure of triptolide caused cardiac dysfunction [7] such as cardiogenic shock, bradyarrhythmia, and acute toxic myocarditis. In previous acute toxicological studies, histological changes of mice heart tissues (myocardial fiber breakage, cell swelling, interstitial congestion, and so on) and the increase of serum cardiac enzyme (LDH, AST, and CK) level indicated that triptolide caused severe myocardial injuries [8–10]. Additionally, *in vitro* experiments demonstrated that triptolide could induce the death of rat cardiomyocytes at nanomolar concentration [11–13]. Although several research groups have reported the cardiotoxicities of triptolide, the underlying mechanisms of triptolide-induced cardiotoxicity (TIC) have not been fully elucidated.

Ferroptosis is a newly identified type of programmed cell death characterized by the iron-dependent accumulation of lipid peroxides to lethal levels [14]. Emerging evidences revealed that ferroptosis plays a critical role in various cardiovascular diseases [15, 16] and drug-induced cardiotoxicities [17–19]. However, the association between ferroptosis and TIC remains unclear. As indicated by its name, iron overload is increasingly recognized as the central mediator of ferroptosis [20]. Excessive iron is engaged in free radical formation, especially reactive oxygen species (ROS), by Fenton reaction which results in the initiation and propagation of the lethal lipid peroxides [21]. Intracellular iron homeostasis is precisely regulated by complicated iron metabolism pathways [22, 23]. Initially, extracellular ferric iron (Fe^3+^) was bound to transferrin (TF) and imported by transferrin receptor 1(TFRC1). As the reduction product of Fe^3+^ in the endosome, ferrous iron (Fe^2+^) is finally released by divalent metal transporter 1 (DMT1, also termed SLC11A2) from endosome to a liable iron pool in the cytoplasm. In normal condition, excessive ferrous iron will be stored in ferritin, an iron storage protein complex including ferritin light chain (FLT) and ferritin heavy chain (FTH). Pathologically, abnormal iron absorption and storage lead to iron overload which promotes the occurrence of lipid peroxidation and the cell vulnerability to ferroptosis.

SLC7A11/GPX4 pathway functions as the canonical defense against ferroptosis by assisting intracellular glutathione (GSH) synthesis and alleviating lipid peroxidation [24]. SLC7A11 (also known as Xct) is a multipass transmembrane protein which mediates the export of intracellular glutamate and import of extracellular cystine at a 1 : 1 ratio [25]. After absorption inside the cell, cystine is reduced to cysteine which serves as the rate-limiting precursor for glutathione (GSH) synthesis [26]. In the presence of GSH, glutathione peroxidase 4 (GPX4) mediates the conversion of toxic lipid peroxides to nontoxic lipid alcohols [27]. SLC7A11 inhibition results in GSH depletion which in turn downregulates GPX4, leading to cellular/subcellular membrane damage caused by accumulation of iron-dependent lipid peroxides [28]. As the pivotal inhibitory pathway of ferroptosis, SLC7A11/GPX4 axis has attracted enormous attention and been targeted as potential treatment of ferroptosis-related diseases.

In the present study, both bioinformatic analysis and experimental validations proved that ferroptosis played an important role in TIC. Furthermore, we explored the involved molecular mechanism and found that the ferroptosis inhibitor ferrostatin-1 (Fer-1) effectively alleviated TIC. These findings provide new insights into the cell death pathways underlying TIC and suggest a novel therapeutic approach for TIC.

## 2. Materials and Methods

### 2.1. Chemicals and Antibodies

Triptolide was purchased from Solarbio Life Science (ST8290, Beijing, China). Triptolide-Biotin were synthesized as described in Supplementary Information. Compounds were dissolved in DMSO at a concentration of 100 mM and stored at -20°C. Ferrostatin-1 (Fer-1) was purchased from Sigma-Aldrich (SML0583, United States). Anti-SLC7A11 rabbit polyclonal antibody (386116) and anti-GLS2 rabbit polyclonal antibody (163996) were purchased from Zen Bio (Chengdu, Sichuan, China). Anti-GPX4 mouse monoclonal antibody (67763-1-Ig) was purchased from Proteintech (Wuhan, Hubei, China). Anti-4-HNE rabbit polyclonal antibody (ab46545) and anti-*β*-actin rabbit polyclonal antibody (ab8227) were purchased from Abcam (Cambridge, UK). Anti-Nrf2 rabbit monoclonal antibody (12721S), anti-HO-1 rabbit monoclonal antibody (5853S), anti-GAPDH rabbit monoclonal antibody (5174S), secondary antibody anti-rabbit IgG (7074S), and anti-mouse IgG (7076S) were purchased from cell signaling technology (Danvers, MA, USA).

### 2.2. Cell Culture

AC16 cells were cultured in Dulbecco's Modified Eagle Medium (DMEM/F12, Gibco) containing 10% fetal bovine serum (FBS, biological industrials (BI)) and 1% penicillin/streptomycin (BI). Cells were maintained in a humidified incubator under 5% CO_2_ at 37°C.

### 2.3. Drug Treatment and Cell Viability Assay

Cell viability was detected using Cell Counting Kit-8 assay (CCK-8, Dojindo, Japan) according to the manufacturer's instruction. AC16 cells were seeded in 96-well plates at a density of 5 × 10^3^ cells per well and incubated for 24 h. At 24 h after seeding, cells were, respectively, treated with 0.1% DMSO (control) and triptolide (20-640 nM) or pretreated with Fer-1 (2 *μ*M) for 2 h before exposed to triptolide (30 nM). The final concentration of DMSO never exceeded 1‰ of the total culture volume. After treatment for 24 h, cells were washed with PBS buffer gently and incubated with DMEM/F12 medium containing 10% CCK-8 solution for 1-3 h. Optical density (OD) value was measured with a microplate reader (Multiskan MK3, Thermo Fisher Scientific, United States) at 450 nm.

Cell survival rate = [(OD value of the experimental group − OD value of the blank group)/(OD value of the control group − OD value of the blank group)] × 100%.

### 2.4. RNA Sequencing and Data Analysis

AC16 cells are treated, respectively, with DMSO (control) and triptolide (30 nM) for 24 h and sent to Novogene Corporation (Beijing, China) for library construction and sequencing. The clustering of the index-coded samples was performed on a cBot Cluster Generation System using TruSeq PE Cluster Kit v3-cBot-HS (Illumia) according to the manufacturer's instructions. After cluster generation, the library preparations were sequenced on an Illumina Nova-seq platform, and 150 bp paired-end reads were generated. In the step of quality data control, clean data were obtained by removing reads containing adapter, reads containing N base, and low quality reads from raw data. All the downstream analyses were based on the clean data with high quality. Differential expression analysis of two conditions/groups (two biological replicates per condition) was performed using the DESeq2 R package (1.20.0). DESeq2 provide statistical routines for determining differential expression in digital gene expression data using a model based on the negative binomial distribution. The resulting *p* values were adjusted using the Benjamini and Hochberg's approach for controlling the false discovery rate. Genes with an adjusted *p* value < 0.05 found by DESeq2 were assigned as differentially expressed. KEGG (Kyoto Encyclopedia of Genes and Genomes) is a database resource for understanding high-level functions and utilities of the biological system, such as the cell, the organism, and the ecosystem, from molecular-level information, especially large-scale molecular datasets generated by genome sequencing and other high-through put experimental technologies (https://www.genome.jp/kegg/). We used clusterProfiler R package to test the statistical enrichment of differential expression genes in KEGG pathways.

### 2.5. Determination of Intracellular Iron Levels

The intracellular iron concentration was detected by the iron ion colorimetric detection kit (E1042, Applygen Technologies, Beijing, China). The AC16 cells were seeded in 24-well plates at a density of 5 × 10^4^ cells per well. At seeding after 24 h, cells were exposed to DMSO (control) and triptolide (30 nM) or pretreated with DFO (50 *μ*M) for 2 h before cotreated with triptolide (30 nM). After treatment for 24 h, cells were washed with cold PBS buffer twice and lysed with RIPA buffer for 2 h. According to the manufacturer's instruction, a standard stock solution was prepared immediately using the dilute solution. Mixture A was prepared by mixing the buffer solution with 4.5% potassium permanganate solution at the ratio of 1 : 1. Next, thoroughly mix the sample with the detection working solution, and incubate at 60°C for 1 h. At the end of the incubation, the iron ion detection reagent was added and incubated at room temperature for 30 minutes. 200 *μ*L of the final solution was added to a 96-well plate per well, and the concentration of iron ions in the cells was detected at 550 nm using a microplate reader (Multiskan MK3, Thermo Fisher Scientific, USA). The intracellular iron levels were then normalized by cell numbers.

### 2.6. Measurement of Malonaldehyde (MDA) and Glutathione Levels

Lipid peroxidation MDA assay kit (S1031S) were purchased from Beyotime (Jiangsu, China). In brief, cells were sonicated in RIPA buffer on ice. Cell lysates were then centrifuged at 12,000 g for 15 min at 4°C to collect the supernatant. MDA levels were measured using a lipid peroxidation MDA assay kit according to the manufacturer's instruction and detected at 532 nm using a microplate reader (Multiskan MK3, Thermo Fisher Scientific, USA). MDA levels were normalized by the protein concentration. GSH levels were measured by a microreduced glutathione (GSH) assay kit (Solarbio Life Science, BC1175, Beijing, China) according to the manufacturer's instruction and detected 412 nm using a microplate reader (Multiskan MK3, Thermo Fisher Scientific, USA). The GSH levels were further normalized by cell numbers.

### 2.7. Measurement of Intracellular ROS Levels

ROS levels was measured by flow cytometry using DCFH-DA probe (S0033S, Beyotime, Jiangsu, China). Cells were treated, respectively, with DMSO (control) and triptolide (7.5, 15, 30, and 60 nM). After treatment for 24 h, cells were collected and incubated in DMEM/F12 medium containing DCFH-DA (10 *μ*M, 20 mins) at 37°C in the dark. Cells were washed with DMEM/F12 medium three times. The samples were then analyzed with a FACStar-Plus flow cytometer (Becton Dickinson, Franklin Lakes, NJ, USA) and the FlowJo (TreeStar) software for acquisition and analysis.

### 2.8. Protein Extraction and Western Blot

After treatment, cells were harvested and lysed with RIPA buffer (C1053, Applygen Technologies, Beijing, China) containing protease/phosphatase inhibitor cocktail (GRF101/102, EpiZyme, Shanghai, China). Protein concentrations were determined using a BCA assay kit (P1511, Applygen Technologies, Beijing, China). Protein samples were separated by SDS-PAGE gels and transferred onto polyvinylidene fluoride membranes (Millipore, Billerica, MA, United States). After blocked with 5% nonfat milk for 1 h, the membranes were incubated successively with specific primary and secondary antibodies. The protein blots were visualized with “Torchlight” Hypersensitive ECL Western HRP Substrate (17046, Zen Bio, Chengdu, China) and automatic exposure system (Image Quant LAS500, GE, Fairfield, CT, USA).

### 2.9. Streptavidin–Biotin Affinity Pull-Down Assay

The biotin-affinity pull-down assay was performed as previously described [29, 30]. Triptolide-biotin were synthesized and verified by ^1^H and ^13^C NMR (see Supplementary Information (available here)). AC16 cells were lysed with RIPA buffer (C1053, Applygen Technologies, Beijing, China) containing protease/phosphatase inhibitor cocktail (GRF101/102, EpiZyme, Shanghai, China) and centrifuged at 12,000 g for 15 min at 4°C to collect the supernatant. The cell lysates were incubated, respectively, with biotin (10 *μ*M), triptolide-biotin (10 *μ*M), and the combination of triptolide (100 *μ*M) and triptolide-biotin (10 *μ*M) for 12 h at 4°C. The magnetic beads (MCE) were then added to the cell lysates and incubated for 4 h at 4°C. The supernatant was removed, and the magnetic beads were boiled in SDS-PAGE sample buffer. The samples were tested by immunoblot with indicated antibodies.

### 2.10. Molecular Docking

The 3D structures of triptolide were drawn by ChemBioDraw Ultra 20.0 and then subjected to energy optimization by the MM2 force field. The 3D structure of 7CCS was downloaded from the PDB (https://www.rcsb.org/pdb/home/home.do) [31, 32]. The ligand and protein files were prepared using AutoDock tools. The protein was optimized by removing water molecules, adding hydrogen, and adding Geister charges, and protein file was saved in PDBQT format. Ligand file was also saved in PDBQT format. The protein-ligand grid map was calculated surrounding active site of protein molecule. Molecular docking analysis were carried out by Autodock Vina. Autodock Vina was run using configuration file and output file generated contained theoretical binding affinity. The lesser binding affinity corresponds to better results. The docking results were visualized using PyMol.

### 2.11. Statistical Analysis

All results were presented as mean ± standard deviation (SD) and generated from at least three independent experiments. Comparison between two or more groups was performed using the Student's *t* test and ANOVA for normal variables or the Mann–Whitney *U* test and Kruskal-Wallis test for nonnormal variables that could not be log transformed (e.g., because of frequent zero values). A value of *p* < 0.05 was chosen as the threshold for statistical significance. Statistical analysis was performed using GraphPad Prism version 8 (GraphPad Software, La Jolla, CA).

## 3. Results

### 3.1. Triptolide Potentially Induces Ferroptosis in AC16 Cells

**Figure 1:**
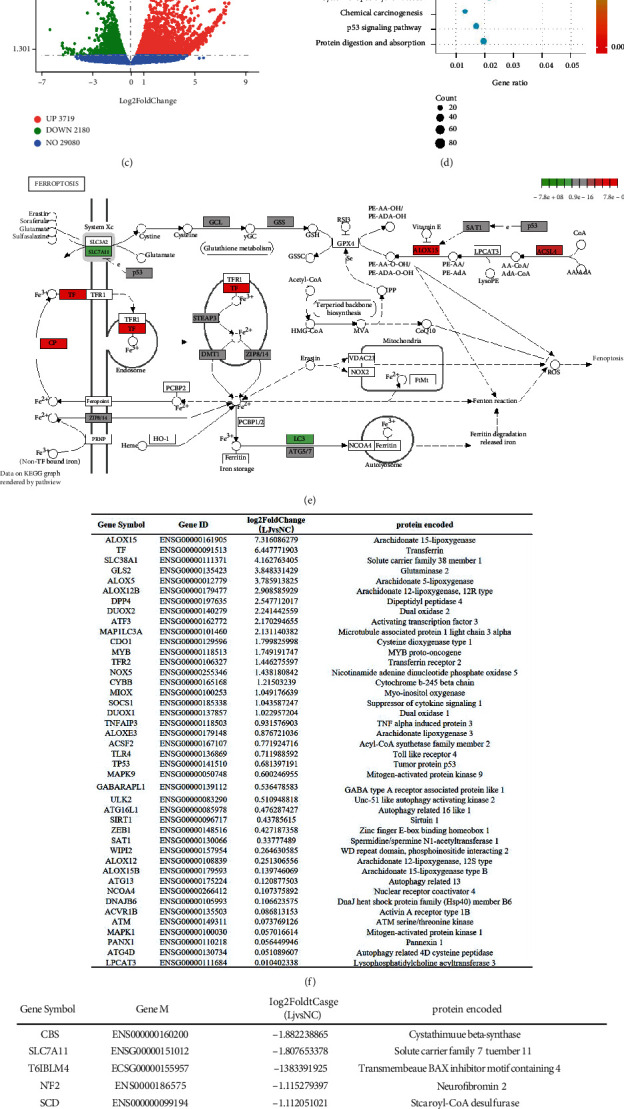
Triptolide induces ferroptosis in AC16 cells indicated by RNA-seq analysis. (a) Chemical structure of triptolide. (b) Cell viability of AC16 cells after treatment with triptolide for 24 h was detected using CCK-8 assay. ns: no significant; ^∗∗∗∗^*p* < 0.0001 vs. the control group. (c) Triptolide-induced differentially enriched genes. (d) Top 20 KEGG pathway with high count. (e) Main targets of triptolide in ferroptosis. (f) DEGs identified as ferroptosis diver in FerrDb. (g) DEGs identified as ferroptosis suppressor in FerDb.

The chemical structure of triptolide is shown in Figure 1(a). To determine the cytotoxicity of triptolide on human cardiomyocytes, AC16 cells were exposed to multiple concentrations of triptolide (0-1280 nM, Figure 1(b)) for 24 h. The CCK-8 results showed that triptolide exhibited significant inhibitory effects on the cell viability of AC16 cells in a dose-dependent manner. To investigate the underlying mechanism of TIC, AC16 cell post triptolide (30 nM) stimulation for 24 h was subjected to the RNA sequencing (RNA-seq) analysis. As shown in the volcano plot (Figure 1(c)), there were a total of 5899 differentially expressed genes (DEGs) (|Fold change| > 2, *p* value < 0.05, 3719 genes upregulated, and 2180 genes downregulated). KEGG enrichment analysis suggested that ferroptosis emerged as the main cell death form in the process of TIC (Figure 1(d)). KEGG graph of ferroptosis (Figure 1(e)) showed that genes related with GSH, iron, and polyunsaturated fatty acid metabolism, such as SLC7A11, TF, and ACSL4, were involved in the process. Furthermore, we overlapped DEGs with genes identified as ferroptosis driver and suppressor in ferroptosis database FerrDb [33]. DEGs which change trends were in coincidence with ferroptosis driver and suppressor were, respectively, listed in Figures 1(f) and 1(g), including lipid peroxidation related-genes (ALOX15, ALOX5, ALOX12, NOX5, DUOX1, and DUOX2), glutathione metabolism genes (SLC7A11, GLS2, and GPX4), and iron metabolism genes (TF, FTH1, NCOA4, and MAP1LC3A). Overall, these results suggested a role of ferroptosis in the pathogenesis of TIC.

### 3.2. Triptolide Provokes Lipid Peroxidation, Excess Iron Accumulation, and GSH Depletion

**Figure 2:**
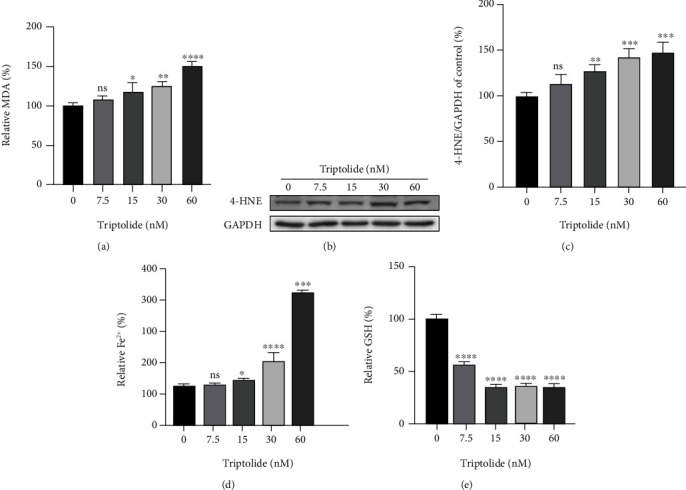
Triptolide provokes lipid peroxidation, excess iron accumulation, and GSH depletion. (a) MDA levels after treatment with triptolide for 24 h were detected by commercial assay kits. (b, c) The protein levels of 4-HNE were measured by Western blots. (d) Intracellular Fe^2+^ levels in AC16 cells. (e) Reduced GSH levels in AC16 cells. ns: no significant; ^∗^*p* < 0.05, ^∗∗^*p* < 0.01, ^∗∗∗^*p* < 0.001, ^∗∗∗∗^*p* < 0.0001 vs. the control group.

To validate the role of ferroptosis in TIC, we assessed the key features of ferroptosis including lipid peroxidation, excess iron accumulation, and GSH depletion [34]. As the most prevalent byproduct of lipid peroxidation, MDA levels increased dose-dependently after treatment with triptolide for 24 h (Figure 2(a)). Additionally, Western blot results showed that the expression of 4-HNE, a classic indicator of ferroptosis-induced lipid hydroperoxides, was gradually elevated as the increase of triptolide concentration (Figures 2(b) and 2(c)). Moreover, the accumulation of intracellular ferrous iron was induced significantly upon the stimulation of triptolide (Figure 2(d)). Contrary to iron levels, GSH, as the most abundant intracellular antioxidant, decreased dramatically in the presence of triptolide (Figure 2(e)). Collectively, these data validate that ferroptosis is involved in TIC.

### 3.3. Triptolide Triggers Iron Overload through Dysregulating Iron Absorption and Storage System

**Figure 3:**
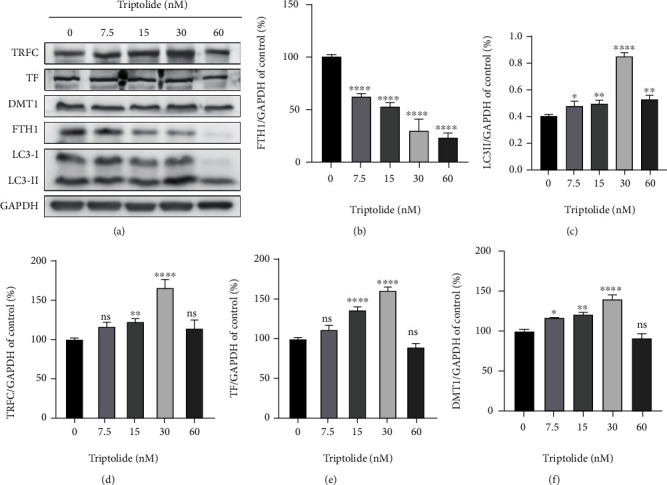
Triptolide triggers iron overload through dysregulating iron absorption and storage system. (a) The protein levels of TRFC, TF, DMT1, FTH1, and LC3-II in AC16 cells were measured by Western blots. (b–f) The quantitative analysis of the indicated proteins. ns: no significant; ^∗^*p* < 0.05, ^∗∗^*p* < 0.01, ^∗∗∗^*p* < 0.001, ^∗∗∗∗^*p* < 0.0001 vs. the control group.

As the central mediator of ferroptosis, cumulative ferrous iron produces excess ROS by Fenton reaction to rapidly amplify lipid peroxides [14, 15]. To unveil the intrinsic molecular mechanism of iron overload, we assessed the expression of genes associated with intracellular iron storage and extracellular iron absorption. After treatment with triptolide (0-60 nM), the expression of FTH1 decreased dose-dependently, paralleling a significant increase of LC3II/I ratio, indicating ferritin was degraded to release ferrous iron in an autophagic manner [35, 36] (Figures 3(a)–3(c)). Besides, Western blot results showed that the expression of TF, TRFC, and DMT1, the genes in charge of iron import, was upregulated in a dose-dependent way after treatment with triptolide (0-30 nM) (Figures 3(d)–3(f)). However, an unexpected decrease of these iron import proteins was detected when AC16 cells were treated with an even higher concentration of triptolide (60 nM).

### 3.4. Triptolide Aggravates ROS Accumulation and Inhibited Nrf2/HO-1 Pathway

**Figure 4:**
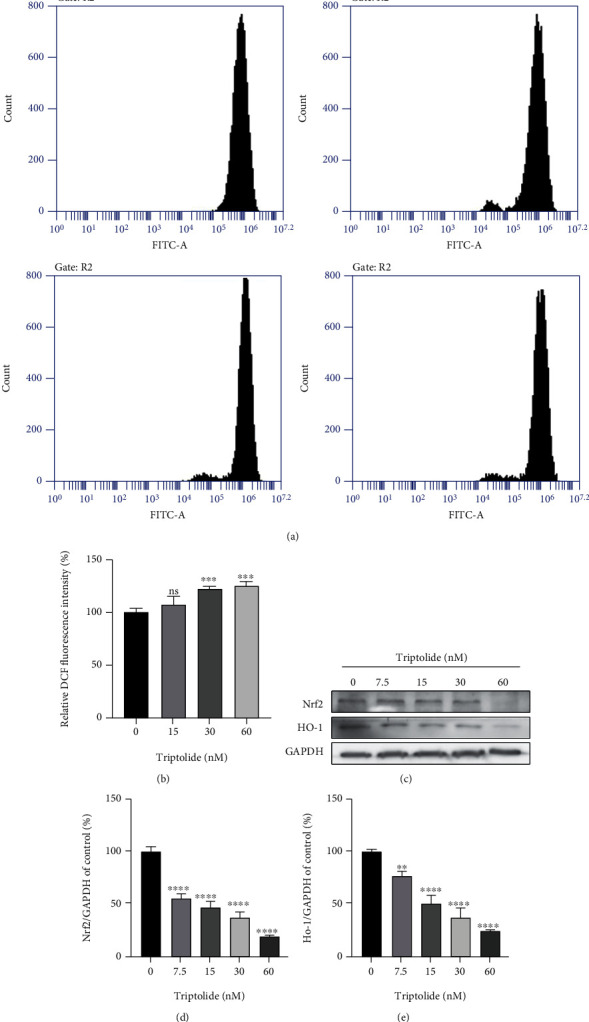
Triptolide aggravated ROS accumulation *via* inhibiting Nrf2/HO-1 pathway. (a, b) ROS levels were assessed by flow cytometry using DCFH-DA probe. (c) The protein levels of SLC7A11 and GPX4 were measured by Western blots. (d, e) The quantitative analysis of the indicated proteins. ns: no significant; ^∗^*p* < 0.05, ^∗∗^*p* < 0.01, ^∗∗∗^*p* < 0.001, ^∗∗∗∗^*p* < 0.0001 vs. the control group.

As increased level of ROS is often accompanied by iron overload, we examined the intracellular ROS levels of AC16 cells with triptolide (0-60 nM) for 24 h. Flow cytometry using DCFH-DA probe showed that triptolide promoted ROS levels significantly in a dose-dependent way (Figures 4(a) and 4(b)). As Nrf2/HO-1 is considered as the classic antioxidant pathway participating in ferroptosis regulation [37], we next assessed the alteration of Nrf2/HO-1 signal axis. Western blot results showed that the expression of Nrf2 and HO-1 decreased significantly as the raise of triptolide treatment concentration, suggesting the inhibitory effects of triptolide on Nrf2/HO-1 pathway exacerbated ROS accumulation in human cardiomyocytes (Figures 4(c)–4(e)), which succumbed cells to ferroptosis through generation of excess lipid peroxides.

### 3.5. Triptolide Downregulates SLC7A11/GPX4 Axis by Direct Binding to SLC7A11

**Figure 5:**
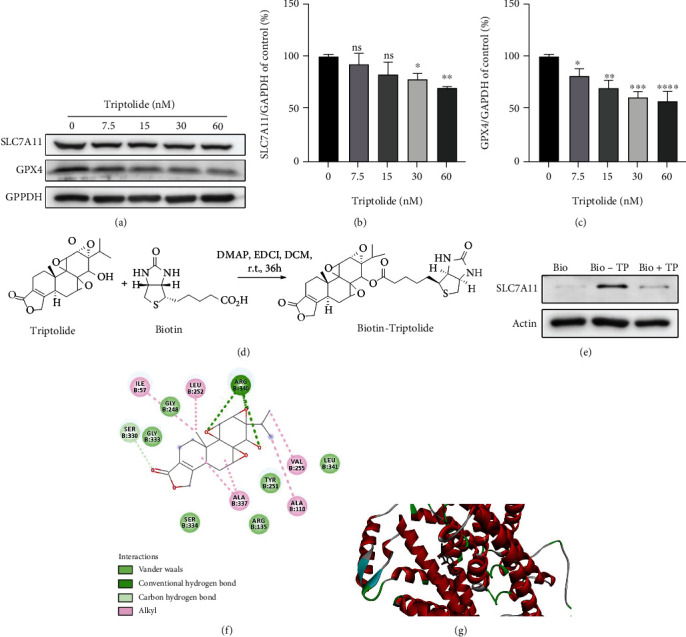
Triptolide downregulates SLC7A11/GPX4 axis by direct binding to SLC7A11. (a) The protein levels of SLC7A11 and GPX4 were measured by Western blots. (b, c) The quantitative analysis of the indicated proteins. (d) The synthesis procedure of biotin-triptolide. (e) Immunoblot with anti-SLC7A11 antibody of protein precipitated by streptavidin beads from AC16 cell lysates in the presence of biotin-triptolide (10 *μ*M), biotin (10 *μ*M) or the combination of triptolide (100 *μ*M), and biotin-triptolide (10 *μ*M). (f) Predicted binding sites of triptolide with SLC7A11. (g) Representations of the predicted binding mode of triptolide with SLC7A11. ns: no significant; ^∗^*p* < 0.05, ^∗∗^*p* < 0.01, ^∗∗∗^*p* < 0.001, ^∗∗∗∗^*p* < 0.0001 vs. the control group.

SLC7A11/GPX4 axis is recognized as the primary defense mechanism against ferroptosis due to its potent function to deoxify excess lipid peroxides with the help of GSH. Therefore, we explored the expression of SLC7A11/GPX4 axis after treatment with triptolide for 24 h. As shown in Figures 5(a)–5(c), SLC7A11/GPX4 axis was remarkably inhibited in response to triptolide treatment. Furthermore, to investigate the direct target by which triptolide inhibited SLC7A11/GPX4 axis, we labelled triptolide with biotin through esterification reaction (Figure 5(d)). Triptolide was incubated with streptavidin beads and AC16 cell lysates. Western blot results indicated that SLC7A11 is a bona fide binding partner of triptolide in triptolide-treated AC16 cells (Figure 5(e)). This result was further validated by molecular docking. As shown in the modeling, molecular docking [38, 39] also demonstrated triptolide interacted with the amino acid residues on SLC7A11 by van der Waals, carbon hydrogen bond, and conventional hydrogen bond (Figures 5(f) and 5(g)). These data supported that the directing binding of triptolide to upstream signaling protein SLC7A11 mediates the downregulation of SLC7A11/GPX4 axis by triptolide.

### 3.6. Fer-1 Attenuates TIC by Restoring SLC7A11/GPX4 Axis

**Figure 6:**
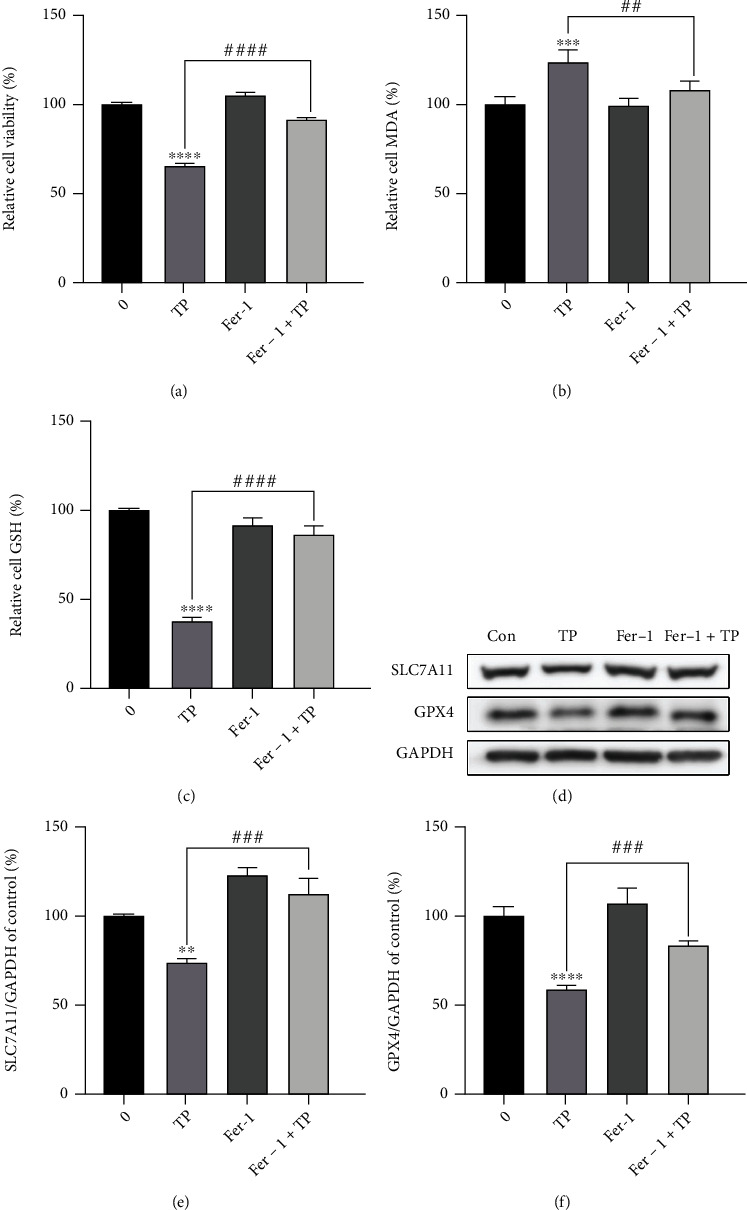
Fer-1 attenuates TIC by restoring SLC7A11/GPX4 axis. (a–d) AC16 cells were pretreated with or without fer-1 (2 *μ*M) for 1 h and then exposed to triptolide (30 nM) for another 24 h. (a) Cell viability of AC16 cells were detected using CCK8 assays. (b) MDA levels were detected by commercial assay kits. (c) Reduced GSH levels in AC16 cells. (d) The protein levels of SLC7A11 and GPX4 were measured by Western blots. (e, f) The quantitative analysis of the indicated proteins. ^∗∗∗∗^*p* < 0.0001 vs. the control group, *^##^p* < 0.01, *^###^p* < 0.001, *^####^p* < 0.0001 vs. the triptolide group.

Since ferroptosis was demonstrated to be involved in the pathogenesis of TIC, we next explored whether the ferroptosis inhibitor Fer-1 could serve as a potential therapeutic method. As shown in Figures 6(a) and 6(b), Fer-1 significantly rescued triptolide-induced cell death and attenuated corresponding lipid peroxidation. Additionally, intracellular GSH deprivation was ameliorated in the presence of Fer-1 (Figure 6(c)). To gain insights into how Fer-1 scavenged lipid hydroperoxyl radicals, we subsequently investigated the effect of Fer-1 on SLC7A11/GPX4 axis. As shown in Figures 6(d)–6(f), the inhibitory effects of triptolide on SLC7A11/GPX4 axis was partially reversed by Fer-1. Thus, our results suggest Fer-1 as a potential remedy to TIC alleviated ferroptosis through upregulation of the SLC7A11/GPX4 axis to eliminate excess lipid peroxides.

## 4. Discussion

Triptolide, as the major active component isolated from *Tripterygium wilfordii* Hook F, has attracted enormous interests from pharmaceutical industries and researchers due to its unique activities on rheumatoid arthritis, leukemia, solid tumors, HIV, and autoimmune diseases [2–4]. However, its clinical application is strongly restricted by severe side effects, especially cardiotoxicity. Emerging evidences have validated that triptolide induces severe cardiotoxicities both *in vivo* and *in vitro*, whereas the underlying mechanism has not been fully elucidated. The major findings from the present study unveiled that ferroptosis, an innovative cell death mode, contributed to TIC. Furthermore, our data suggested that triptolide enhanced intracellular ferrous iron accumulation by reducing iron storage and increasing iron absorption. Excess iron induced ROS accumulation which is further aggravated by the inhibition of Nrf2/HO-1 antioxidant pathway in triptolide-treated AC16 cells. ROS resulted in the overproduction of lethal lipid peroxides. Meanwhile, the direct inhibitory effects of triptolide on SLC7A11 led to GSH depletion and cascade downregulation of GPX4 which collectively disrupted the clearance of lipid peroxidation and exacerbated ferroptosis. Finally, we found that the ferroptosis inhibitor Fer-1 conferred cardioprotection against TIC through reversing the expression of SLC7A11/GPX4 axis. These findings clarified the molecular events underlying TIC and indicated Fer-1 as a potential therapeutic method for TIC.

Ferroptosis is a nonapoptotic, newly recognized cell death pattern caused by iron-dependent accumulation of lipid peroxidation. Recent studies have demonstrated the vital role of ferroptosis in various cardiovascular diseases and drug-induced cardiotoxicities, such as ischemia/reperfusion (I/R)- and doxorubicin-induced cardiac injuries [40]. In this study, RNA-seq and KEGG analysis indicated that ferroptosis may participate in TIC, as related genes involving lipid and iron metabolism enriched significantly. Consistently, we found that triptolide caused lipid peroxidation as indicated by the increase of MDA and 4-HNE levels, iron overload as indicated by rising ferrous iron levels, and GSH depletion as indicated by descending reduced GSH levels. As the key events of ferroptosis, these data further validated that ferroptosis is involved in TIC. Ferroptosis is triggered once the balance between the generation and the clearance of lipid peroxidation is disrupted [41]. Iron overload contributes to the initiation and amplification of lipid peroxides through producing ROS by Fenton reaction. The liable iron pool in the cytoplasm is precisely controlled by an iron import and storage system. Extracellular iron was transported inside with the help of TF/TFRC/DMT1 pathway. The excess iron is stored in the form of ferritin under normal conditions. After triptolide treatment (0-30 nM) for 24 h, the expression of TF, TRFC, and DMT1 increased significantly in a dose-dependent manner, while the expression of FTH1 showed an opposite trend. Upregulation of LC3-II/I ratio indicated the autophagic degradation of FTH1. Interestingly, although the expression of FTH1 continued to decrease at a higher concentration of triptolide (60 nM), an unexpectedly reduced expressions of TF, TRFC, and DMT1 were observed compared with triptolide stimulation at a concentration of 30 nM. We thought this phenomenon may act as the compensatory mechanism to antagonize the progressively higher level of intracellular iron.

Nrf2/HO-1 pathway, a classic antioxidant pathway, has been proved as a defensive response against ferroptosis in many studies. Emerging as a potential target, activation of Nrf2/HO-1 pathway has shown to be an effective way to attenuate ferroptosis-induced injuries and diseases, including LPS-induced acute lung injury [42], contusion spinal cord injury [43], type 2 diabetic osteoporosis [44], and radiation-induced intestinal epithelial cell death [45]. In our hand, we found that triptolide showed potent inhibition on Nrf2/HO-1 pathway, which disrupted the elimination of ROS. ROS accumulation driven by iron overload and further exacerbated by inactivation of Nrf2/HO-1 antioxidant pathway synergistically resulted in the excess generation of lethal lipid peroxides. These results were in line with the previous findings [42–47].

Reduced GSH is an essential intracellular antioxidant synthesized from glutamate, cysteine, and glycine, which works together with GPX4 to converts toxic lipids to nontoxic lipids [48]. The rate of glutathione synthesis is limited by cysteine availability. SLC7A11 mediates the import of extracellular cystine that is to be converted to cysteine by GSH or TXNRD1. The inhibition of SLC7A11 will lead to GSH exhaustion thus directly impacting on GPX4 activity and stability and thereby predisposing cells to ferroptosis. In the streptavidin-biotin affinity pull-down assay, triptolide exhibited direct and strong affinity with SLC7A11. Computational molecular docking indicated that triptolide could interact with amino acid residues on SLC7A11 by van der Waals, carbon hydrogen bond, conventional hydrogen bond, and alkyl. In addition, triptolide inhibited the expression of SLC7A11 and GPX4. Taken together, triptolide led to exhaustion of GSH and deteriorated the clearance of lethal lipid peroxides through direct inhibition on SLC7A11 and cascade downregulation of GPX4. Fer-1, as the ferroptosis inhibitor, has been extensively investigated in various ferroptosis-related diseases. Cumulative evidences suggested Fer-1 attenuated ferroptosis by targeting at SLC7A11/GPX4 pathway. In this study, the application of Fer-1 potently rescued triptolide-induced cytotoxicity and reversed ferroptosis-related events including the accumulations of MDA iron. In-depth studies unveiled that the downregulation of SLC7A11 and GPX4 could be alleviated by Fer-1. These data indicated Fer-1 as a potential therapeutic approach to TIC.

## 5. Conclusion

**Figure 7:**
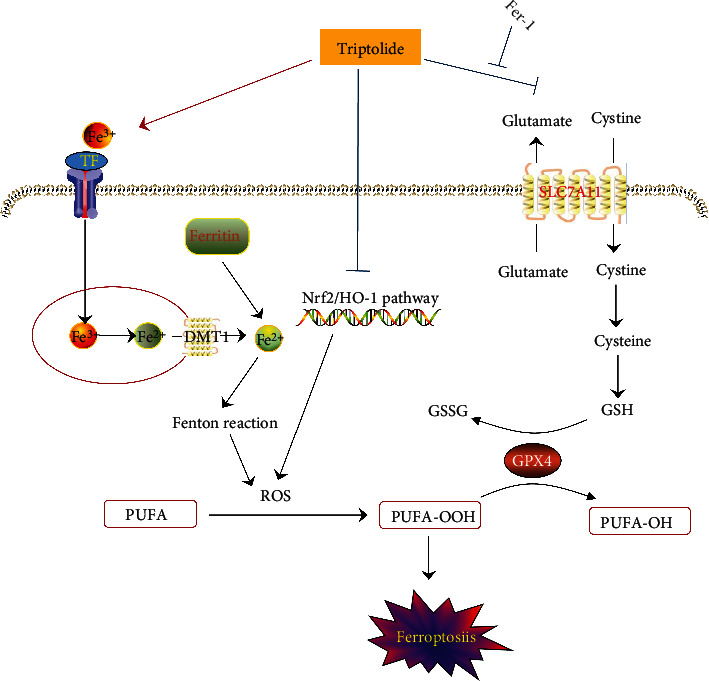
Schematic representation of the potential toxic mechanism involved in triptolide-induced ferroptosis.

Our current study found that TIC was causatively associated with ferroptosis. As shown in Figure 7, triptolide induced iron overload by dysregulation of iron metabolism which led to ROS accumulation through Fenton reaction. Meanwhile, triptolide impeded ROS elimination *via* inactivation of Nrf2/HO-1 antioxidant system. Excess ROS triggered the overproduction of lethal lipid peroxides. In addition, triptolide-induced GSH depletion hindered the clearance of lipid peroxides and promoted ferroptosis by direct inhibition on SLC7A11 and cascade downregulation on GPX4. Fortunately, Fer-1 could alleviate triptolide-induced ferroptosis through restoring SLC7A11/GPX4 pathway. In summary, our study provided a new insight into the underlying mechanisms of TIC and presented a potential therapeutic strategy for circumventing this side effects.

Triptolide induced intracellular iron overload through dysregulating TF/TFRC/DMT1/FTH1 pathway which mediated iron absorption and storage. Excess iron contributed to ROS generation via Fenton reaction. The inhibitory effects of triptolide on antioxidant Nrf2/HO-1 pathway exacerbated ROS accumulation which led to overproduction of lethal lipid peroxides. Furthermore, triptolide directly bound to SLC7A11 to inactivate SLC7A11/GPX4 axis which functioned to detoxify of toxic lipid peroxides to nontoxic lipid alcohols. Fer-1 alleviated triptolide-induced ferroptosis via partially reversing the inhibitory effects of triptolide on SLC7A11/GPX4 signal.

## Data Availability

The data used to support the findings of this study are included within the article and supporting information.

## Supplementary Materials

Supplementary Materials A detailed description of the synthesis method of triptolide-biotin and its related spectra were provided in the Supporting Information.
