# Supplementary material for: SLC7A11/GPX4 Inactivation-Mediated Ferroptosis Contributes to the Pathogenesis of Triptolide-Induced Cardiotoxicity
Source: Oxid Med Cell Longev. 2022 Jun 16;2022:3192607. doi: 10.1155/2022/3192607 (PMC9225845; doi:10.1155/2022/3192607)
Supplement: Supplementary Materials — A detailed description of the synthesis method of triptolide-biotin and its related spectra were provided in the Supporting Information. [file 3192607.f1.docx]

**Supporting Information**

1. **General Information**

Unless otherwise noted, all commercially available chemicals including solvents were used without further purification. D-Biotin (E080117), 1-(3-dimethylaminopropyl)-3-ethylcarbodiimide hydrochloride (EDCI, A0109380250), 4-dimethylaminopyridine (DMAP, B040170), ethyldiisopropylamine (DIPEA, W3200140250) and dichloromethane (DCM, W6101541000) were purchased from Energy Chemical (Shanghai, China). ^1^H NMR, ^13^C NMR were recorded on a Bruker ADVANCE III (400 MHz) spectrometer with CDCl_3_ as the solvent and tetramethylsilane (TMS) as the internal standard.

1. **Synthesis of Biotin-Triptolide**

D-Biotin (82 mg, 0.335 mmol, 2.0 equiv.), EDCI (96.5 mg, 0.503mmol, 3.0 equiv.), and DMAP (61.5 mg, 0.503mmol, 3.0 equiv.), were dissolved in DCM (5.0 mL) under the protection of nitrogen. Triptolide (60 mg, 0.166 mmol, 1.0 equiv.) and DIPEA (130.0 mg, 1.006 mmol, 6.0 equiv.) was dissolved in DCM (1.0 mL) and added to the above mixture. After stirring overnight at room temperature, the resulting mixture was quenched by 1 N hydrochloric acid solution and extracted with ethyl acetate (EtOAc) three times. The combined organic phase was washed with brine, dried over anhydrous Na2SO4, and concentrated in vacuo after filtration. The desired biotin-triptolide was obtained as white solid by silica-gel flash column chromatography (EtOAc/petroleum ether = 1:1). ^1^H NMR (400 MHz, CDCl_3_) δ 5.81 (s, 1H), 5.09 (d, *J* = 7.8 Hz, 2H), 4.68 (s, 2H), 4.59- 4.45 (m, 1H), 4.41- 4.26 (m, 1H), 3.85 (d, *J* = 2.7 Hz, 1H), 3.52 (dd, *J* = 18.2, 3.8 Hz, 2H), 3.15 (dd, *J* = 11.5, 6.9 Hz, 1H), 2.90 (dd, *J* = 12.9, 4.7 Hz, 1H), 2.73 (dd, *J* = 28.5, 12.8 Hz, 2H), 2.49 (t, *J* = 7.3 Hz, 2H), 2.33 (d, *J* = 16.4 Hz, 1H), 2.17 (dd, *J* = 13.1, 7.3 Hz, 2H), 1.96- 1.85 (m, 2H), 1.73- 1.66 (m, 2H), 1.63- 1.46 (m, 3H), 1.29- 1.22 (m, 1H), 1.05 (s, 3H), 0.96 (d, *J* = 6.9 Hz, 3H), 0.84 (d, *J* = 6.8 Hz, 3H). ^13^C NMR (101 MHz, CDCl3) δ 173.22, 173.06, 159.94, 125.64, 70.82, 69.99, 63.75, 63.39, 61.83, 61.34, 60.11, 59.84, 55.74, 55.65, 54.89, 40.77, 40.38, 35.71, 33.78, 29.91, 28.13, 24.78, 23.45, 17.57, 17.08, 16.76, 13.82. HRMS (ESI): calcd for [C_30_H_38_N_2_O_8_S+ H^+^]: 587.2427, found: 587.2431.

1. **^1^H NMR spectrum of Biotin-Triptolide**


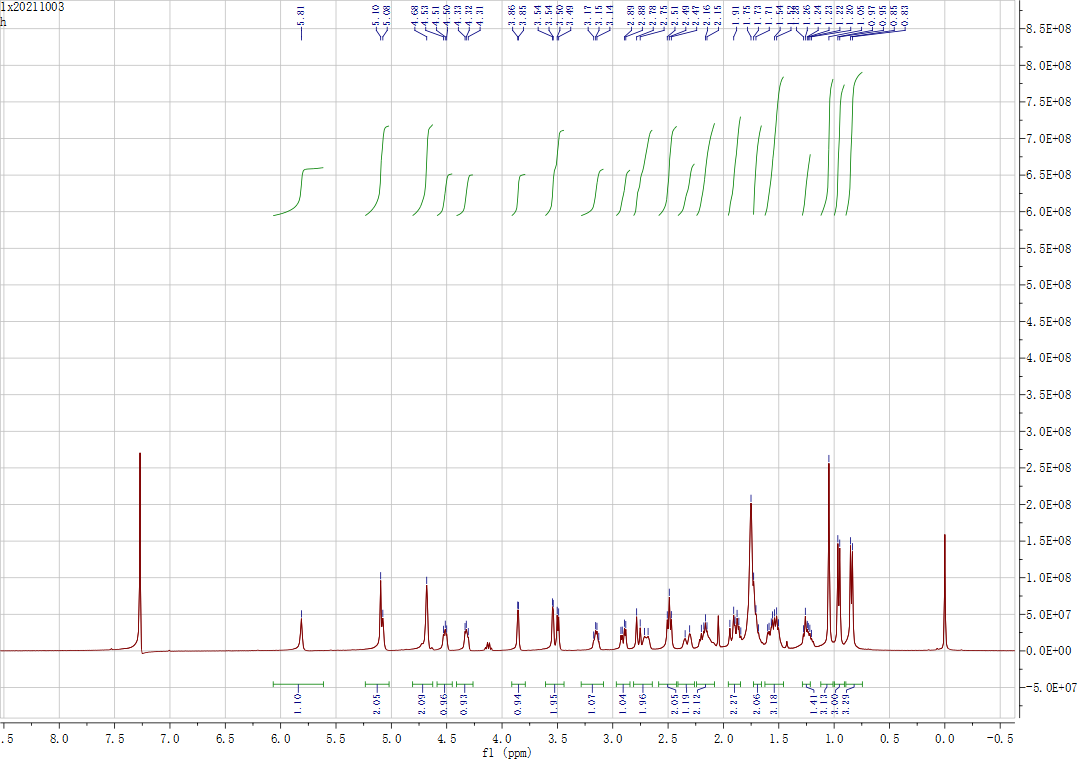


1. **^13^C NMR spectrum of Biotin-Triptolide**

**
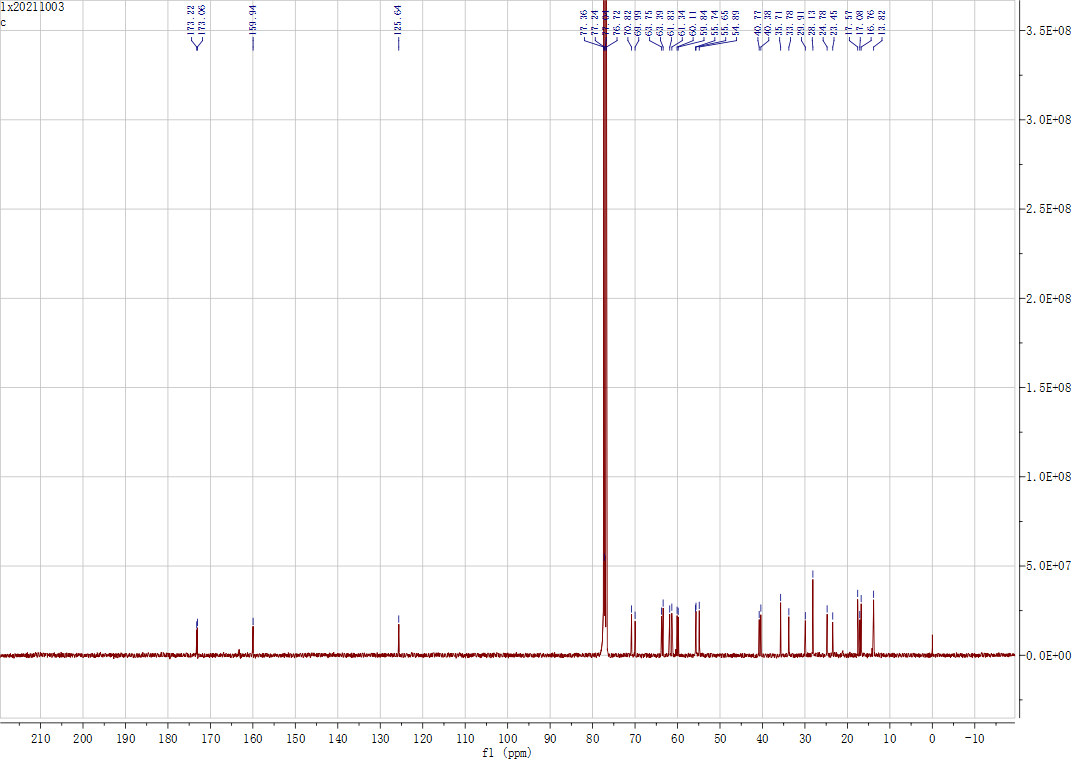
**

1. **HRMS spectrum of Biotin-Triptolide**
